# Mental disorders, mortality following myocardial infarction, and the impact of the COVID-19 pandemic in England: a cohort study

**DOI:** 10.1093/ehjqcco/qcag044

**Published:** 2026-03-17

**Authors:** Kelly Fleetwood, John Nolan, Colin Berry, Debbie Cavers, Stewart W Mercer, Sandosh Padmanabhan, Daniel J Smith, Robert Stewart, Amanda Vettini, Caroline A Jackson

**Affiliations:** Usher Institute, University of Edinburgh, 5-7 Little France Road, Edinburgh BioQuarter ‒ Gate 3, Edinburgh EH16 4UX, UK; British Heart Foundation Data Science Centre, Health Data Research UK, London NW1 2BE, UK; School of Cardiovascular and Metabolic Health, University of Glasgow, Glasgow G12 8TA, UK; Usher Institute, University of Edinburgh, 5-7 Little France Road, Edinburgh BioQuarter ‒ Gate 3, Edinburgh EH16 4UX, UK; Usher Institute, University of Edinburgh, 5-7 Little France Road, Edinburgh BioQuarter ‒ Gate 3, Edinburgh EH16 4UX, UK; School of Cardiovascular and Metabolic Health, University of Glasgow, Glasgow G12 8TA, UK; Institute of Neuroscience and Cardiovascular Research, University of Edinburgh, Edinburgh EH16 4SB, UK; Department of Psychological Medicine, King’s College London, London SE5 8AF, UK; South London and Maudsley NHS Foundation Trust, London SE5 8AZ, UK; Usher Institute, University of Edinburgh, 5-7 Little France Road, Edinburgh BioQuarter ‒ Gate 3, Edinburgh EH16 4UX, UK; Usher Institute, University of Edinburgh, 5-7 Little France Road, Edinburgh BioQuarter ‒ Gate 3, Edinburgh EH16 4UX, UK

## Abstract

**Aims:**

People with schizophrenia experience poorer cardiovascular disease (CVD) outcomes, but other mental disorders have been little studied. We compared post-myocardial infarction (MI) mortality by mental disorder, explored whether differences in care contributed to mortality disparities, and investigated whether disparities worsened during the COVID-19 pandemic.

**Methods and results:**

We identified people with MI in England (November 2019—February 2023) from the Myocardial Ischaemia National Audit Programme, ascertaining mental disorder diagnoses from linked electronic health records and mortality from national death records. We used logistic regression to compare 30-day and one-year mortality between people with each of schizophrenia, bipolar disorder, or depression (of any severity) vs. those without these disorders for ST-elevation MI (STEMI) and non-STEMI (NSTEMI), adjusting for confounders and investigating differences by calendar time period. For one-year mortality, we additionally adjusted for receipt of guideline-informed care. We included 131 075 patients with NSTEMI and 79 045 with STEMI. For NSTEMI, schizophrenia [odds ratio (OR) 1.73, 95% CI 1.20–2.49] and depression (1.17, 1.08–1.26) were associated with higher 30-day mortality. For STEMI, 30-day mortality was higher in people with schizophrenia (1.61, 1.09–2.37), bipolar disorder (1.68, 1.08–2.59), and depression (1.10, 1.01–1.20). All disorders were associated with higher one-year mortality following NSTEMI and STEMI, with adjustment for care attenuating estimates. Relative mortality disparities were generally unaffected by the COVID-19 pandemic.

**Conclusion:**

Our findings highlight the increased risk of post-MI mortality in people with mental disorders. Improved implementation of acute cardiac care standards may help to close the gap.

## Structured Graphical Abstract

**Structured Graphical Abstract:**
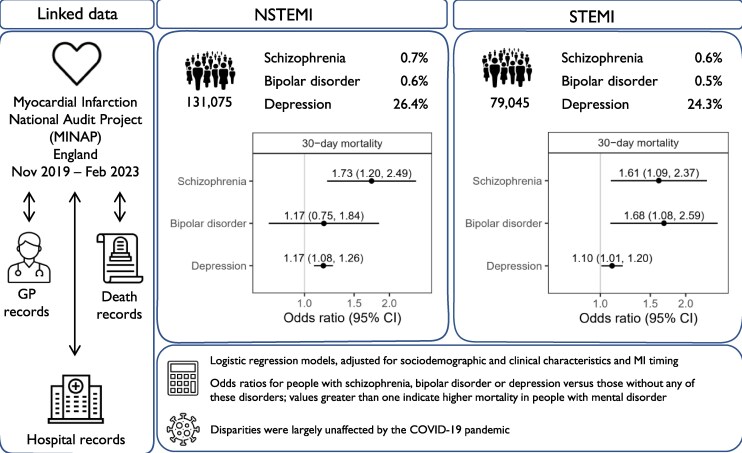

Key Learning Points What is already known

- Mental disorders are associated with an elevated risk of, and poor outcomes from, cardiovascular disease.
- Studies have demonstrated that following myocardial infarction (MI), people with mental disorders have higher 30-day and one-year mortality.
- However, existing studies have largely focused on schizophrenia, rarely differentiated by MI type and have not examined the effect of the COVID-19 pandemic.

What this study adds

- Our study of over 200 000 patients in England identified mortality disparities for both NSTEMI and STEMI, which were greatest for people with schizophrenia, but also evident for bipolar disorder and depression.
- Our findings suggest that differences in receipt of care may contribute to poorer survival at one year and that disparities were generally unaffected by the COVID-19 pandemic.
- Our study highlights the urgent need to better understand why people with schizophrenia, bipolar disorder, or depression experience disparities in post-MI mortality and to develop approaches to improve survival in this marginalized group.

## Introduction

Mental disorders are associated with an elevated risk of, and poor outcomes from, cardiovascular disease (CVD).^1,2^ Studies from both universal and non-universal healthcare settings have demonstrated that following myocardial infarction (MI), people with mental disorders have higher 30-day and one-year mortality,^3^ a disparity that persists in recent decades.^4,5^ Previous studies have largely focused on schizophrenia,^5–10^ with little investigation of other conditions such as bipolar disorder and depression.^4,11,12^ Analysis by type of MI is scarce and limited to non-universal healthcare settings.^13–15^ Given differences in clinical management and survival of NSTEMI and STEMI, mental disorder-related outcome disparities may differ by MI type. While the mechanisms underlying the observed MI mortality disparities remain poorly understood, these are likely multifactorial^2^ and may include differences in receipt of acute MI care, with studies consistently finding a lower receipt of percutaneous coronary intervention (PCI) in people with a mental disorder.^3^ We recently found that, in England, people diagnosed with a mental disorder had lower receipt of clinical guideline-informed care standards for NSTEMI, including receipt and timeliness of angiogram, admission to cardiac ward, receipt of secondary prevention medication prescribing on discharge and cardiac rehabilitation referral.^16^ We also found some differences in receipt of care standards for STEMI, such as timeliness of revascularization, although patterns varied by mental disorder and care standard. However, the contribution of disparities in clinical care to MI survival by mental disorder status remains little investigated, with inconsistent findings from studies that adjusted mental disorder-mortality associations for receipt of revascularization.^3,7,17^

Furthermore, the response to the COVID-19 pandemic led to significant health care delivery disruption, including reduced cardiovascular care activity.^18–20^ Studies also revealed greater delays from symptom onset to seeking medical help for ischaemic heart disease (IHD) and increased short-term mortality during the early period of the pandemic compared to the pre-pandemic period.^21^ To the best of our knowledge, no study has examined whether the pandemic exacerbated existing mental disorder disparities in MI survival.

In this study, for each of NSTEMI and STEMI, we sought to: (i) compare our primary outcome, 30-day mortality, and secondary outcomes including one-year mortality among people with vs. without schizophrenia, bipolar disorder or depression of any severity; (ii) explore the extent to which disparities in receipt of care might explain mortality disparities; and (iii) determine whether mortality disparities worsened during the COVID-19 pandemic.

## Methods

We have reported this study in accordance with the Reporting of Studies Conducted using Observational Routinely-Collected Health Data (RECORD) statement.^22^

### Setting and data sources

We accessed national linked electronic health records from England in NHS England’s (NHSE) Secure Data Environment (SDE), made available through the British Heart Foundation Data Science Centre’s CVD-COVID-UK/COVID-IMPACT Consortium.^23^ Our primary data source was the Myocardial Ischaemia National Audit Project (MINAP), a clinical audit of patients admitted to hospital with MI, which includes information on patient demographics, medical history, clinical characteristics of the MI, and care from the call for help to discharge.^24^ We also used data from the General Practice Extraction Service (GPES) Data for Pandemic Planning and Research (GDPPR) dataset, which captures data from 98% of GP practices in England, and includes diagnoses from the primary care domain reference set (see Supplementary material online, *Methods S1*).^25^ The GDPPR dataset includes patients who are currently registered and deceased patients with a date of death on or after 1 November 2019. For patients in the GDPPR dataset, available records included diagnoses up to 31 July 2024. GP records transfer between practices when a patient moves, and so should capture an individual’s entire medical history. We also used the Hospital Episode Statistics Admitted Patient Care (HES APC) dataset, which included inpatient admissions and day cases from 1 April 1997 to 31 July 2024. The HES APC dataset includes the primary diagnosis (main reason for hospitalization), up to 19 secondary diagnoses and up to 24 procedures. Data from the Civil Registrations of Death, which includes all deaths registered in England, was available from 1 November 2019 to 31 July 2024, and we extracted the date of death and the underlying cause of death. Finally, we also used data from COVID-19 Hospitalisations in England Surveillance System (CHESS), Second Generation Surveillance System (SGSS), and Secondary Uses Service (SUS), all of which can be used to identify people with COVID-19 infection.

### Study design and study population

We conducted a retrospective cohort study, including all adults (≥ 18 years of age) with a final diagnosis of NSTEMI or STEMI recorded in MINAP between 1 November 2019 and 28 February 2023 (diagnostic criteria for NSTEMI and STEMI are included in Supplementary material online, *Methods S2*). We did not include events prior to 1 November 2019, since GDPPR data were unavailable for people who died before this date. We excluded patients without a linked GDPPR record and those who failed quality assurance checks. For each individual, we included the first record of an MI within the study period.

### Mental disorder

We ascertained history of schizophrenia, bipolar disorder, and depression (of any severity) from the GDPPR and HES APC datasets, based on diagnoses at any time prior to each individual’s MI admission date. For people with diagnoses of more than one disorder, we categorized them according to their most severe condition, with schizophrenia considered the most severe, followed by bipolar disorder and depression. For the HES APC dataset, we used ICD-10 codes, as per previous research,^4,26^ to identify schizophrenia (F20, F25), bipolar disorder (F30, F31), and depression (F32, F33) from the primary and secondary diagnosis fields. For the GDPPR dataset, we identified each disorder using SNOMED codes (see Supplementary material online, *Methods S1*). The comparison group included people without a diagnosis of any of these mental disorders prior to their MI admission date.

### Outcomes

Our primary outcome was 30-day mortality, measured from the MI admission date. We also evaluated: one-year mortality; one-year mortality amongst patients who survived 30 days (in order to evaluate whether any differences in one-year mortality were due to differences within and/or beyond the first 30 days); time to mortality within the first year; and time to CVD mortality within the first year. We defined CVD mortality based on deaths where the underlying cause was IHD (ICD-10 codes I20-I25) or cerebrovascular disease (I60-I69, G45). Finally, we evaluated one-year mortality among patients discharged home, adjusting for receipt of guideline-indicated hospital care indicators recommended for all such patients.

### Covariates

We ascertained age, sex, deprivation (based on quintiles of the Index of Multiple Deprivation,^27^ a neighbourhood-level measure of deprivation), and ethnicity from the CVD-COVID-UK/COVID-IMPACT Consortium key patient characteristics table.^28^ We defined MI admission timing variables: daytime or overnight; weekday or weekend and the 4-month period during which the admission occurred; and identified the hospital where the patient was admitted (see Supplementary material online, *Methods S2*). We identified history of MI and comorbid angina, cerebrovascular disease, chronic renal failure, diabetes, and heart failure from the MINAP dataset and from diagnoses recorded in GDPPR or HES APC prior to the date of MI admission, based on SNOMED and ICD-10 code lists^4,23,29–33^ (see Supplementary material online, *Methods S2*). We identified history of PCI from the MINAP dataset and from procedures recorded in HES APC using an existing OPCS-4 code list^4^ (see Supplementary material online, *Methods S2*). We ascertained MI presentation features, including cardiac arrest, cardiogenic shock, ST deviation, creatinine within the first 24 h after admission (μmol/L), heart rate on admission, and the first systolic blood pressure (SBP) (mmHg) measurement after admission from the MINAP dataset. We identified patients who had COVID-19 within 14 days either side of their MI (see Supplementary material online, *Methods S2*). We also defined guideline-informed care standards relevant to patients discharged home, specifically receipt of angiography and admission to cardiac ward for NSTEMI, receipt of primary PCI and call-to-balloon (CTB) time (i.e. time from call for help to receipt of primary PCI) within target time for STEMI, and indicated secondary prevention medication prescribed at discharge and referral for cardiac rehabilitation for both types of MI (see Supplementary material online, *Methods S2*).

### Statistical analyses

We analysed NSTEMI and STEMI separately, using mixed effects logistic regression for binary outcomes and mixed effects Cox proportional hazards models for time-to-event outcomes. For time to CVD mortality within the first year, we accounted for the competing risk of death from other causes by censoring people who died from other causes at their date of death. We obtained odds ratios (ORs) (for binary outcomes) and hazard ratios (HRs) (for time-to-event outcomes), comparing patients with each of schizophrenia, bipolar disorder or depression to those without any of these disorders.

For each of NSTEMI and STEMI, we serially adjusted for groups of potential confounding factors: model 1 adjusted for age and sex, with random effects for the intercept for each hospital; model 2 additionally adjusted for ethnicity and deprivation; model 3 additionally adjusted for timing variables; model 4 additionally adjusted for clinical history (history of MI, history of PCI and comorbidities); and model 5 additionally adjusted for MI presentation features (cardiac arrest, cardiogenic shock, creatinine, heart rate, SBP and COVID-19 status for both types of MI, and additionally ST deviation for NSTEMI only) (see Supplementary material online, *Methods S3*). For one-year mortality amongst patients discharged home, we additionally sequentially adjusted for guideline-informed care standards. Age, creatinine, heart rate, and SBP were included in the models as continuous variables. We scaled age, SBP, and heart rate by subtracting their respective means and dividing by their standard deviations. Creatinine was right-skewed and hence was log-transformed prior to scaling to improve convergence of the models. For all continuous variables, we included both linear and quadratic terms in the models because exploratory plots suggested that quadratic functions were sufficient for describing the relationships between these variables and the outcomes. For the mixed effects Cox proportional hazards models, we used log cumulative hazard plots to check the proportional hazards assumption.

We investigated whether any associations between mental disorder and mortality were affected by the COVID-19 pandemic. Given that our study data were only available from November 2019, we evaluated the effect of the pandemic by comparing the associations in the pre-pandemic period (November 2019—February 2020) to the associations in each of the following 4-month periods. We categorized the time period into four-month intervals because we wanted to capture fluctuations in mortality throughout our study period without making prior assumptions that specific periods (e.g. periods with high COVID-19 mortality) would have higher or lower mortality rates. In addition to periods with high COVID-19 mortality, mortality throughout our study period may have been affected by lockdowns and periods of increased demand on ambulance services and hospitals. For the analysis of the impact of the COVID-19 pandemic, we used a binary mental disorder variable (any of schizophrenia, bipolar disorder, or depression vs. none of these disorders) since there were insufficient numbers to investigate the interaction for individual mental disorders. We refitted the final model for each outcome using the binary mental disorder variable instead of the original categorical variable and fitted a further model including the interaction between the binary mental disorder variable and the 4-month time period. We examined whether the interaction term improved model fit using a likelihood ratio test.

The primary analyses excluded patients with missing data in the outcome or any of the covariates. Missing data were minimal amongst the sociodemographic, timing, and clinical history covariates, but higher amongst the MI presentation covariates. Hence, we also conducted a *post hoc* sensitivity analysis that omitted the MI presentation covariates from the sequence of models and excluded only patients with missing data in this narrower set of covariates.

In accordance with statistical disclosure control rules, counts are rounded to the nearest 5, and counts less than 10 are suppressed. We used R (version 4.1.3 and above)^34^ to conduct the statistical analysis, the package lme4^35^ to fit mixed effects logistic regression models, and the package coxme^36^ to fit mixed effects Cox proportional hazards models. All R code is available in our GitHub repository (https://github.com/BHFDSC/CCU046_02).

## Results

### Descriptive findings

**Figure 1:**
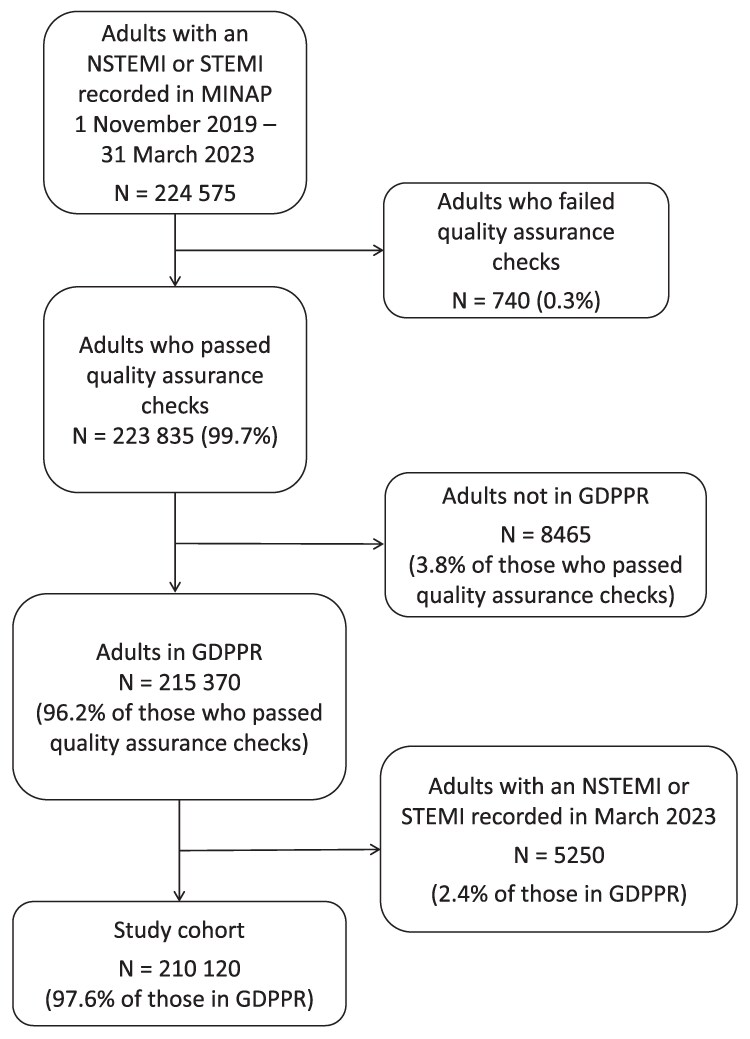
Flow diagram for the study cohort.

Our study included 131 075 patients with NSTEMI and 79 045 with STEMI (*Figure 1*, Supplementary material online, *Figure S1*). Among patients with NSTEMI, 875 (0.7%), 750 (0.6%), and 34 610 (26.4%) had a prior record of schizophrenia, bipolar disorder, and depression, respectively, with prevalence similar among patients with STEMI.

For both NSTEMI and STEMI, people with vs. without a mental disorder diagnosis were: younger at MI onset; more likely to be women; and more likely to live in deprived areas. Minority ethnic groups were over-represented among patients with schizophrenia, and under-represented among patients with bipolar disorder or depression, relative to the comparison group. People with a mental disorder diagnosis were more likely to be admitted overnight and generally had a higher prevalence of CVD, cerebrovascular disease, and diabetes. MI presentation features were broadly similar across groups (*Tables 1* and *2*).

**Table 1:** Baseline characteristics of patients with a MINAP record of an NSTEMI, by mental disorder status^a^

Characteristic	Schizophrenia (*n* = 875)	Bipolar disorder (*n* = 750)	Depression (*n* = 34 610)	None of these disorders (*n* = 94 840)
**Sociodemographics**				
Age at MI (years), mean (SD)	64.7 (12.5)	65.3 (12.9)	67.5 (13.0)	70.6 (13.3)
Female (%)	295 (33.7)	320 (42.7)	15 170 (43.8)	27 840 (29.4)
IMD decile (%)				
1 (most deprived)	195 (22.3)	120 (16.0)	5210 (15.1)	9710 (10.2)
2	155 (17.7)	105 (14.0)	4155 (12.0)	9150 (9.6)
3	115 (13.1)	75 (10.0)	3830 (11.1)	9290 (9.8)
4	105 (12.0)	105 (14.0)	3655 (10.6)	9570 (10.1)
5	70 (8.0)	65 (8.7)	3470 (10.0)	9620 (10.1)
6	65 (7.4)	70 (9.3)	3275 (9.5)	9805 (10.3)
7	45 (5.1)	75 (10.0)	3100 (9.0)	9535 (10.1)
8	55 (6.3)	45 (6.0)	2870 (8.3)	9425 (9.9)
9	35 (4.0)	55 (7.3)	2645 (7.6)	9210 (9.7)
10 (least deprived)	30 (3.4)	40 (5.3)	2380 (6.9)	9410 (9.9)
Missing	<10 (<1.1)	<10 (<1.3)	25 (0.1)	110 (0.1)
Ethnicity				
Black	30 (3.4)	10 (1.3)	365 (1.1)	1700 (1.8)
South Asian	125 (14.3)	50 (6.7)	1785 (5.2)	7990 (8.4)
White	680 (77.7)	675 (90.0)	31 535 (91.1)	81 330 (85.8)
Mixed	<10 (<1.1)	<10 (<1.3)	240 (0.7)	685 (0.7)
Other ethnic groups	30 (3.4)	10 (1.3)	675 (2.0)	2970 (3.1)
Missing	<10 (<1.1)	<10 (<1.3)	10 (<0.1)	170 (0.2)
**Location and timing**				
NHS trust				
Available	855 (97.7)	725 (96.7)	33 660 (97.3)	92 260 (97.3)
Missing	20 (2.3)	25 (3.3)	955 (2.8)	2580 (2.7)
Admission day				
Week day	670 (76.6)	565 (75.3)	26 030 (75.2)	71 370 (75.3)
Weekend	205 (23.4)	190 (25.3)	8580 (24.8)	23 470 (24.7)
Admission timing				
Daytime	485 (55.4)	425 (56.7)	20 815 (60.1)	58 720 (61.9)
Overnight	390 (44.6)	330 (44.0)	13 795 (39.9)	36 120 (38.1)
**Clinical history**				
Previous MI (%)	235 (26.9)	215 (28.7)	9220 (26.6)	21 755 (22.9)
Previous PCI (%)	200 (22.9)	210 (28.0)	10 525 (30.4)	27 310 (28.8)
Angina (%)	420 (48.0)	390 (52.0)	17 200 (49.7)	42 710 (45.0)
Cerebrovascular disease (%)	225 (25.7)	195 (26.0)	7105 (20.5)	14 980 (15.8)
Chronic renal failure (%)	100 (11.4)	85 (11.3)	3455 (10.0)	9750 (10.3)
Diabetes (%)	430 (49.1)	320 (42.7)	13 780 (39.8)	33 475 (35.3)
Heart failure (%)	300 (34.3)	215 (28.7)	10 030 (29.0)	25 700 (27.1)
**MI presentation features**				
ST deviation (%)	170 (19.4)	130 (17.3)	6585 (19.0)	19 320 (20.4)
Missing (%)	30 (3.4)	15 (2.0)	1140 (3.3)	2810 (3.0)
Cardiogenic shock (%)	<10 (<1.1)	<10 (<1.3)	125 (0.4)	410 (0.4)
Missing (%)	115 (13.1)	75 (10.0)	3820 (11.0)	10 310 (10.9)
Cardiac arrest (%)	35 (4.0)	15 (2.0)	755 (2.2)	2445 (2.6)
Missing (%)	25 (2.9)	10 (1.3)	660 (1.9)	1880 (2.0)
Creatinine (μmol/L)				
Median [IQR]	83.0 [68.0, 104.0]	83.0 [68.0, 104.0]	82.0 [68.0, 101.0]	85.0 [72.0, 105.0]
Missing (%)	35 (4.0)	20 (2.7)	970 (2.8)	2475 (2.6)
SBP (mmHg)				
Mean (SD)	138.2 (27.5)	140.0 (28.2)	143.3 (27.3)	145.1 (27.4)
Missing (%)	35 (4.0)	15 (2.0)	795 (2.3)	2340 (2.5)
Heart rate (bpm)				
Mean (SD)	85.8 (20.4)	82.8 (19.8)	80.7 (19.0)	79.8 (19.2)
Missing (%)	30 (3.4)	15 (2.0)	830 (2.4)	2420 (2.6)
COVID-19 positive (%)	50 (5.7)	45 (6.0)	1715 (5.0)	4995 (5.3)

bpm, beats per minute; IMD, index of multiple deprivation; IQR, interquartile range; MI, myocardial infarction; PCI, percutaneous coronary intervention; SBP, systolic blood pressure; SD, standard deviation.

^a^As per the statistical disclosure control rules, counts are rounded to the nearest 5 and counts less than 10 are suppressed.

**Table 2:** Baseline characteristics of patients with a MINAP record of a STEMI, by mental disorder status^a^

	Schizophrenia (*n* = 490)	Bipolar disorder (*n* = 405)	Depression (*n* = 19 230)	None of these disorders (*n* = 58 920)
**Sociodemographics**				
Age at MI (years), mean (SD)	60.3 (13.1)	61.6 (13.0)	63.6 (12.8)	66.5 (13.3)
Female (%)	130 (26.5)	170 (42.0)	7200 (37.4)	13 995 (23.8)
IMD decile (%)				
1 (most deprived)	95 (19.4)	65 (16.0)	3040 (15.8)	6280 (10.7)
2	85 (17.3)	45 (11.1)	2450 (12.7)	5930 (10.1)
3	70 (14.3)	50 (12.3)	2180 (11.3)	5800 (9.8)
4	60 (12.2)	45 (11.1)	2055 (10.7)	5995 (10.2)
5	45 (9.2)	40 (9.9)	1860 (9.7)	5980 (10.1)
6	30 (6.1)	20 (4.9)	1750 (9.1)	5970 (10.1)
7	30 (6.1)	40 (9.9)	1590 (8.3)	5855 (9.9)
8	30 (6.1)	50 (12.3)	1560 (8.1)	5925 (10.1)
9	25 (5.1)	25 (6.2)	1495 (7.8)	5745 (9.8)
10 (least deprived)	15 (3.1)	20 (4.9)	1240 (6.4)	5405 (9.2)
Missing	<10 (<2.0)	<10 (<2.5)	10 (0.1)	45 (0.1)
Ethnicity				
Black	15 (3.1)	<10 (<2.5)	180 (0.9)	905 (1.5)
South Asian	50 (10.2)	15 (3.7)	825 (4.3)	4950 (8.4)
White	390 (79.6)	375 (92.6)	17 710 (92.1)	50 640 (85.9)
Mixed	10 (2.0)	<10 (<2.5)	125 (0.7)	425 (0.7)
Other ethnic groups	20 (4.1)	<10 (<2.5)	360 (1.9)	1750 (3.0)
Missing	<10 (<2.0)	<10 (<2.5)	30 (0.2)	250 (0.4)
**Location and timing**				
NHS trust (%)				
Available	485 (99.0)	390 (96.3)	18 945 (98.5)	58 045 (98.5)
Missing	<10 (<2.0)	15 (3.7)	285 (1.5)	875 (1.5)
Admission day				
Week day	355 (72.4)	305 (75.3)	13 810 (71.8)	42 220 (71.7)
Weekend	135 (27.6)	100 (24.7)	5420 (28.2)	16 700 (28.3)
Admission timing				
Daytime	265 (54.1)	225 (55.6)	11 550 (60.1)	36 845 (62.5)
Overnight	225 (45.9)	180 (44.4)	7675 (39.9)	22 075 (37.5)
**Clinical history**				
Previous MI (%)	85 (17.3)	65 (16.0)	3035 (15.8)	6905 (11.7)
Previous PCI (%)	315 (64.3)	285 (70.4)	14 715 (76.5)	44 735 (75.9)
Angina (%)	130 (26.5)	120 (29.6)	5885 (30.6)	14 830 (25.2)
Cerebrovascular disease (%)	80 (16.3)	80 (19.8)	2565 (13.3)	5815 (9.9)
Chronic renal failure (%)	20 (4.1)	25 (6.2)	815 (4.2)	2340 (4.0)
Diabetes (%)	190 (38.8)	145 (35.8)	6075 (31.6)	15 530 (26.4)
Heart failure (%)	180 (36.7)	115 (28.4)	6265 (32.6)	18 655 (31.7)
**MI presentation features**				
Cardiogenic shock (%)	25 (5.1)	15 (3.7)	580 (3.0)	2130 (3.6)
Missing (%)	75 (15.3)	40 (9.9)	2700 (14.0)	8150 (13.8)
Cardiac arrest (%)	60 (12.2)	50 (12.3)	2290 (11.9)	7630 (12.9)
Missing (%)	15 (3.1)	10 (2.5)	355 (1.8)	1330 (2.3)
Creatinine (micromole/L)				
Median [IQR]	80.0 [67.0, 99.0]	78.0 [64.2, 99.0]	78.0 [65.0, 94.0]	81.0 [69.0, 98.0]
Missing (%)	30 (6.1)	20 (4.9)	1230 (6.4)	3310 (5.6)
SBP (mmHg)				
Mean (SD)	129.3 (27.2)	128.8 (26.3)	132.7 (27.7)	133.0 (27.7)
Missing (%)	30 (6.1)	15 (3.7)	755 (3.9)	2410 (4.1)
Heart rate (bpm)				
Mean (SD)	85.2 (20.8)	82.0 (19.5)	80.1 (19.3)	79.0 (19.3)
Missing	25 (5.1)	10 (2.5)	680 (3.5)	2210 (3.8)
COVID-19 positive (%)	25 (5.1)	15 (3.7)	835 (4.3)	2595 (4.4)

bpm, beats per minute; IMD, index of multiple deprivation; IQR, interquartile range; MI, myocardial infarction; PCI, percutaneous coronary intervention; SBP, systolic blood pressure; SD, standard deviation.

^a^As per the statistical disclosure control rules, counts are rounded to the nearest 5 and counts less than 10 are suppressed.

### Mental disorder and 30-day and one-year mortality

Among NSTEMI patients, 6600 (5.0%) died within 30 days, with the crude percentage highest among those with schizophrenia. Following a STEMI, 7440 (9.4%) patients died within 30 days, with the crude percentages highest amongst those with schizophrenia or bipolar disorder. For both MI types, the proportion of patients who died within one year was greater among those with schizophrenia and bipolar disorder than in the comparison group (*Table 3*).

**Table 3:** Mortality and receipt of care among people with each of NSTEMI and STEMI, by mental disorder status^a^

Mortality	Schizophrenia	Bipolar disorder	Depression	None of these disorders
**NSTEMI**				
All patients (*N*)	875	750	34 610	94 840
30-day all-cause mortality (%)	60 (6.9)	35 (4.7)	1625 (4.7)	4875 (5.1)
1-year all-cause mortality (%)	165 (18.9)	115 (15.3)	4540 (13.1)	12 915 (13.6)
1-year CVD mortality (%)	85 (9.7)	65 (8.7)	2320 (6.7)	6305 (6.6)
Patients who survived 30 days (*N*)	815	715	32 990	89 960
1-year all-cause mortality (%)	100 (12.3)	80 (11.2)	2920 (8.9)	8035 (8.9)
Patients discharged home (*N*)	660	535	26 210	71 420
1-year all-cause mortality (%)	100 (15.2)	70 (13.1)	2805 (10.7)	7700 (10.8)
Angiogram (%)				
Received within 72 h	185 (28.0)	200 (37.4)	10 590 (40.4)	29 835 (41.8)
Received out with 72 h	155 (23.5)	135 (25.2)	6805 (26.0)	18 660 (26.1)
Received, timing unknown	30 (4.5)	25 (4.7)	1485 (5.7)	3925 (5.5)
Eligible, but no receipt	155 (23.5)	85 (15.9)	3240 (12.4)	8495 (11.9)
Not eligible	110 (16.7)	65 (12.1)	3275 (12.5)	8465 (11.9)
Missing	30 (4.5)	20 (3.7)	815 (3.1)	2045 (2.9)
Referred for cardiac rehabilitation (%)				
Yes	420 (63.6)	385 (72.0)	20 265 (77.3)	55 435 (77.6)
No	80 (12.1)	45 (8.4)	2125 (8.1)	5845 (8.2)
Not indicated	85 (12.9)	50 (9.3)	1910 (7.3)	5075 (7.1)
Patient declined	20 (3.0)	10 (1.9)	380 (1.4)	820 (1.1)
Missing	55 (8.3)	40 (7.5)	1535 (5.9)	4250 (6.0)
Discharged on secondary prevention (%)				
Yes	535 (81.1)	440 (82.2)	21 830 (83.3)	60 200 (84.3)
No	100 (15.2)	80 (15.0)	3630 (13.8)	9410 (13.2)
Missing	25 (3.8)	15 (2.8)	750 (2.9)	1810 (2.5)
Admission to cardiac ward (%)				
Yes	260 (39.4)	210 (39.3)	11 110 (42.4)	30 810 (43.1)
No	380 (57.6)	305 (57.0)	14 500 (55.3)	38 925 (54.5)
Missing	20 (3.0)	15 (2.8)	600 (2.3)	1685 (2.4)
**STEMI**				
All patients (*N*)	490	405	19 230	58 920
30-day all-cause mortality (%)	60 (12.2)	50 (12.3)	1705 (8.9)	5625 (9.5)
1-year all-cause mortality (%)	90 (18.4)	70 (17.3)	2570 (13.4)	8190 (13.9)
1-year CVD mortality (%)	65 (13.3)	50 (12.3)	1820 (9.5)	5970 (10.1)
Patients who survived 30 days (N)	430	350	17 520	53 300
1-year all-cause mortality (%)	35 (8.1)	15 (4.3)	860 (4.9)	2565 (4.8)
Patients discharged home (N)	380	330	16 300	49 795
1-year all-cause mortality (%)	30 (7.9)	20 (6.1)	930 (5.7)	2820 (5.7)
Call-to-balloon				
<120 min	105 (27.6)	60 (18.2)	4165 (25.6)	13 370 (26.9)
120–150 min	55 (14.5)	65 (19.7)	3320 (20.4)	9970 (20.0)
≥150 min	105 (27.6)	115 (34.8)	4955 (30.4)	14 530 (29.2)
Received PCI, but CTB time unknown	35 (9.2)	30 (9.1)	1295 (7.9)	4240 (8.5)
Did not receive PCI	80 (21.1)	55 (16.7)	2495 (15.3)	7465 (15.0)
Missing	<10 (<2.6)	<10 (<3.0)	75 (0.5)	225 (0.5)
Referred for cardiac rehabilitation (%)				
Yes	320 (84.2)	290 (87.9)	14 805 (90.8)	44 980 (90.3)
No	25 (6.6)	15 (4.5)	490 (3.0)	1730 (3.5)
Not indicated	20 (5.3)	<10 (<3.0)	335 (2.1)	1075 (2.2)
Patient declined	10 (2.6)	<10 (<3.0)	100 (0.6)	265 (0.5)
Missing	10 (2.6)	15 (4.5)	575 (3.5)	1745 (3.5)
Discharged on secondary prevention (%)				
Yes	340 (89.5)	305 (92.4)	15 085 (92.5)	46 205 (92.8)
No	25 (6.6)	15 (4.5)	805 (4.9)	2525 (5.1)
Missing	15 (3.9)	<10 (<3.0)	410 (2.5)	1065 (2.1)

^a^In accordance with NHSE’s SDE statistical disclosure control rules, counts are rounded to the nearest 5, and counts less than 10 are suppressed.

**Figure 2:**
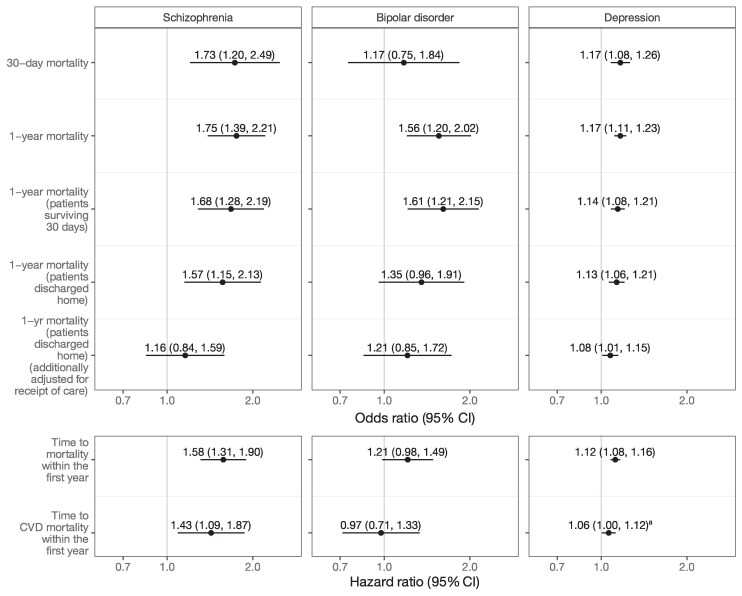
Odds ratios and hazard ratios for mortality following NSTEMI in patients with schizophrenia, bipolar disorder, or depression vs. those without any of these disorders. Ratios are adjusted for sociodemographic characteristics, hospital, MI timing, comorbidities, and MI presentation features (model 5). ^a^1.062 (1.004, 1.123).

After adjusting for sociodemographic and timing covariates, clinical history, and MI presentation features, relative to the comparison group, odds of 30-day mortality following NSTEMI were 73% higher in people with schizophrenia (OR 1.73, 1.20–2.49) and marginally higher in those with depression (1.17, 1.08–1.26). For bipolar disorder, there was no statistically significant difference in 30-day mortality (OR 1.17, 0.75–1.84) with wide confidence intervals reflecting fewer patients in this group (*Figure 2*; Supplementary material online, *Figure S2*).

**Figure 3:**
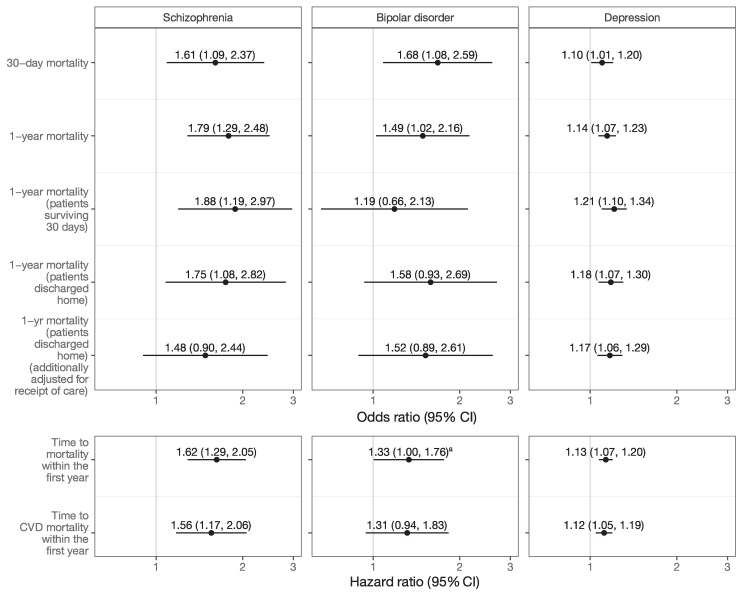
Odds ratios and hazard ratios for mortality following NSTEMI in patients with schizophrenia, bipolar disorder, or depression vs. those without any of these disorders. Ratios are adjusted for sociodemographic characteristics, hospital, MI timing, comorbidities, and MI presentation features (model 5). ^a^1.331 (1.005, 1.762).

For STEMI, after adjustment for all covariates, 30-day mortality was higher for people with schizophrenia (OR 1.61, 1.09–2.37) and bipolar disorder (OR 1.68, 1.08–2.59) and marginally higher for people with depression (OR 1.10, 1.01–1.20) (*Figure 3*; Supplementary material online, *Figure S3*).

For both MI types, each mental disorder was associated with excess odds of one-year mortality after adjustment for all covariates. Among NSTEMI patients, schizophrenia and bipolar disorder were associated with increases of 75% (OR 1.75, 1.39–2.21) and 56% (OR 1.56, 1.20–2.02), respectively, while depression was associated with a more modest increase of 17% (OR 1.17, 1.11–1.23) (*Figure 2*). ORs for the association between mental disorder and one-year mortality following STEMI were similar to those for NSTEMI (*Figure 3*; Supplementary material online, *Figures S2* and *S3*).

Disparities in one-year mortality were not entirely attributable to disparities within the first 30 days. Among NSTEMI patients who survived 30 days, fully adjusted models still indicated higher odds of one-year mortality for people with each of the disorders, relative to the comparison group (*Figure 2*; Supplementary material online, *Figure S2*). Amongst STEMI patients who survived 30 days, the fully adjusted models indicated higher odds of one-year mortality for people with schizophrenia or depression, but not for bipolar disorder, where confidence intervals were extremely wide (*Figure 3*; Supplementary material online, *Figure S3*).

For time to mortality within 1 year, based on the fully adjusted models and relative to the comparison group, survival time was lower in NSTEMI patients with schizophrenia or depression, and in STEMI patients with any of schizophrenia, bipolar disorder, or depression (*Figures 2* and *3*; Supplementary material online, *Figures S2* and *S3*).

For both types of MI, based on the fully adjusted models, HRs for cardiovascular death within one year were statistically significantly higher in people with schizophrenia and (to a much lesser extent) depression, relative to the comparison group. For bipolar disorder, there was no clear evidence of a difference (*Figures 2* and *3*; Supplementary material online, *Figures S2* and *S3*).

### Exploration of effect of receipt of care on one-year mortality

Among NSTEMI patients who were discharged home, those with schizophrenia, bipolar disorder, or depression were less likely to receive guideline-indicated care (*Table 3*). In models of one-year mortality, additional adjustment for receipt of care indicators attenuated effect estimates, particularly for schizophrenia (*Figure 2*), where ORs attenuated from 1.57 (95% CI 1.15–2.13) to 1.16 (95% CI 0.84–1.59).

Among STEMI patients who were discharged home, those with schizophrenia, bipolar disorder, or depression were less likely to receive PCI, and those with schizophrenia were less likely to be referred for cardiac rehabilitation and be discharged on all indicated secondary prevention medication. After adjusting one-year mortality for receipt of care, effect estimates for schizophrenia attenuated from OR 1.75 (95% CI 1.08–2.82) to OR 1.48 (95% CI 0.90–2.44). Estimates attenuated only slightly for depression and bipolar disorder (*Figure 3*).

Individual adjustment for care indicators revealed that most of the attenuation of effect estimates arose from adjusting for receipt of angiogram in NSTEMI, and adjusting for receipt of primary PCI and referral for cardiac rehabilitation in STEMI (see Supplementary material online, *Figures S2* and *S3*).

### Sensitivity analyses

Findings from models that included patients with complete information on sociodemographic, timing, and clinical history variables (97% and 98% of all patients eligible for our NSTEMI and STEMI cohorts, respectively) but did not adjust for MI presentation features were similar to those obtained in the primary analysis (see Supplementary material online, *Figures S4* and *S5*).

### COVID-19 and mental disorder differences in outcomes

Within the study period, overall monthly admissions for both NSTEMI and STEMI were lowest at the start of the COVID-19 pandemic, in March and April 2020 (see Supplementary material online, *Figure S6*). For NSTEMI, 30-day mortality was highest from March to April 2020 and from December 2020 to January 2021. For STEMI, 30-day mortality was highest in March 2020, December 2020, and from December 2022 to January 2023. Time trends were similar for patients with and without a mental disorder diagnosis (see Supplementary material online, *Figure S7*). There was no evidence that the associations between mental disorder and mortality varied over our study period, with one exception; for NSTEMI, the association between mental disorder and time to CVD mortality within the first year varied by admission period (see Supplementary material online, *Table S1*). There was a statistically significant difference between the association in the pre-pandemic period (November 2019—February 2020), and the association in one other period (November 2020—February 2021). In this period only, people with schizophrenia, bipolar disorder or depression had a longer time to CVD mortality than the comparison group (see Supplementary material online, *Table S2*). Findings from the sensitivity analysis were similar, with the association between mortality and mental disorder only varying by period for time to CVD mortality among people with NSTEMI (see Supplementary material online, *Tables S3* and *S4*).

## Discussion

We found that 30-day mortality was higher amongst NSTEMI patients with schizophrenia or depression and amongst STEMI patients with schizophrenia, bipolar disorder, or depression, compared to those without these mental disorders, even after accounting for key confounders. Each of the mental disorders was also associated with higher one-year mortality following either NSTEMI or STEMI. The excess mortality was generally largest for schizophrenia, but was also evident for bipolar disorder, and to a lesser degree, depression. Exploratory analyses suggested that differences in receipt of care may partly explain the excess one-year mortality among those with schizophrenia for both NSTEMI and STEMI. Within our study period, 30-day mortality following NSTEMI or STEMI generally peaked in line with the periods of highest mortality from COVID-19. Trends over time were similar for people with and without a mental disorder, hence disparities in MI mortality generally persisted, but did not widen, during and following the COVID-19 pandemic.

### Disparities by mental disorder

The 30-day and one-year mortality effect estimates generally align with previous findings on mental disorders and MI mortality risk from Scotland^4^ and other non-UK universal healthcare settings.^5,6,8,9,12^ Our study adds to existing understanding by demonstrating similar patterns of excess mortality for both NSTEMI and STEMI. The generally higher excess risk of cardiovascular death in people with a mental disorder in our study aligns with reports of elevated risk of cardiovascular events post-MI in people with mental disorders.^4,17^ The finding that disparities are generally largest for schizophrenia is also consistent with previous studies reporting on multiple mental disorders within the same study population.^4,11,12^ Our findings contribute to the neglected study of bipolar disorder and depression^4,11,12^ with respect to MI outcomes, highlighting disparities among people with mood disorders as well as psychotic disorders. The estimates for depression in the present study are smaller than those from studies where depression was ascertained from hospital admission records, which are likely to predominantly capture more severe depression.^4,15^ A recent study supports possible variation in the association between depression and adverse MI outcomes by antidepressant medication prescribing, which potentially acts as a proxy for depression severity.^37^

Despite increasing evidence of MI mortality disparities by mental disorder status, there has been little advancement in understanding the underlying mechanisms. In our study, associations at both 30 days and one year persisted even after accounting for sociodemographic factors, timing of MI, clinical history, and MI presentation features. Whilst the higher prevalence of comorbidities likely increases the risk of poorer outcome from MI in people with a mental disorder,^38^ this may also indirectly operate through receipt of sub-optimal care.^39^ Studies have demonstrated that patients with comorbid diabetes, e.g. (particularly common in people with mental disorders) are less likely to receive optimal MI care.^40^ Our results suggest that, for schizophrenia, differences in receipt of guideline-informed care standards for NSTEMI and STEMI may contribute to lower one-year survival even after taking account of differences in comorbidity prevalence. This is driven by lower access to reperfusion treatment and (for STEMI), lower referral for cardiac rehabilitation. These findings align with studies from Canada and Hong Kong reporting on schizophrenia^7^ and psychotic disorders,^6^ with adjustment for receipt of reperfusion similarly attenuating one-year mortality estimates (although neither study stratified by MI type and focused on psychosis only).^7^ Additional factors, for which we did not have data or that were beyond the scope of the present study, may also contribute to the observed differences in mortality. These include: presence of cardiac arrythmias, for which people with mental disorders are at a higher risk;^41,42^ longer term prescription of cardiovascular secondary prevention medication, which is less likely in those with mental disorders;^43,44^ and initiation (following referral) of cardiac rehabilitation, which may also be less likely in people with mental disorders since factors associated with initiating rehabilitation, such high socioeconomic status and fewer comorbidities, are less common in those with mental disorders.^45–47^ Finally, we did not examine secondary prevention medication adherence post-MI by mental disorder status, which is an understudied area. Whilst not specific to the post-MI period, one moderately sized study from the United States found no evidence that people with schizophrenia were less likely to adhere to statins or angiotensin-converting enzyme inhibitor/angiotensin receptor blocker medications than people without psychiatric illness.^48^

### Impact of the COVID-19 pandemic

Mortality following NSTEMI or STEMI peaked in line with the periods of highest mortality from COVID-19 in the UK.^49^ To the best of our knowledge, this is the first study to have examined the impact of the pandemic on disparities in post-MI mortality by mental disorder, and, somewhat surprisingly, people with a mental disorder were not disproportionately affected.

During the early phase of the pandemic, the peak in 30-day mortality for both NSTEMI and STEMI in the total study population contrasts with an earlier study using MINAP data from January to May 2020, which reported higher 30-day mortality for NSTEMI but lower 30-day mortality for STEMI.^50^ However, various methodological differences, including differing time periods and inclusion criteria, limit comparability and may explain these differing findings on 30-day mortality.

### Strengths and limitations

Strengths of this study include the use of a national and representative MI audit dataset with near-complete coverage.^24^ To the best of our knowledge, it is the first study to evaluate whether the COVID-19 pandemic affected existing mental disorder disparities in MI outcomes. Separate analysis of NSTEMI and STEMI, and by individual mental disorder, enabled a granular investigation of mortality disparities. In particular, this provides valuable insight into post-MI mortality for people with bipolar disorder or depression, which has been little studied to date. Our study minimized information bias through ascertainment of mental disorders from primary care records and through linkage to national records for deaths. We also adjusted for various confounding factors, including sociodemographic factors and key comorbidities that influence MI survival. Finally, our exploratory assessment of the potential contribution of receipt of care standards across the acute care pathway makes a novel contribution to our understanding of the mechanisms underlying the mental disorder disparities in MI survival.

Our study has some limitations. While the linked primary care data allowed better ascertainment of mental disorder, compared to previous studies using only hospital records, we could not robustly determine depression severity, since the most common depression codes recorded in primary care did not indicate severity. We had planned to focus our study on severe mental illness (schizophrenia, bipolar disorder, and major depression), in order to evaluate mortality disparities in people with the most severe mental disorders, however, due to the lack of specificity in primary care depression codes, we widened our definition of depression to include depression of any severity. Our findings for depression may therefore mask any heterogeneity of effect estimates by depression severity. Some misclassification of mental disorder status is likely, since we used a hierarchical approach to assign people with multiple diagnoses to their most severe disorder, and since people may not always seek help from their primary care providers for mental disorders, particularly depression. Furthermore, we did not account for how recently a mental disorder was diagnosed before the MI, so some patients may not have had an active mental disorder at the time of their MI. Although the overall pattern of findings for bipolar disorder was generally similar to that for schizophrenia, effect estimates were less precise for bipolar disorder due to fewer patients with this disorder. Similarly, the smaller number of STEMI events led to reduced precision of effect estimates for mortality outcomes for bipolar disorder and schizophrenia. We performed complete case analysis rather than conducting multiple imputation, given that 80% of cases were complete for the primary analysis and over 97% were complete in the sensitivity analyses where we excluded the MI presentation characteristics. Findings were similar for both the primary and the sensitivity analyses. Our estimates of mortality disparities may be residually confounded by lifestyle factors such as smoking, which is associated with post-MI mortality^51^ and has a prevalence of approximately 40% in those with a mental disorder vs. 20% in those without, based on general population data from England.^52^ Body mass index (BMI) may also contribute to residual confounding in our estimates of mortality disparities since it is higher, on average, in people with vs. without mental disorders. Likewise, the attenuation of mortality disparities, once we adjusted for receipt of care standards, may also reflect residual confounding. Whilst the linked MINAP, hospital, and primary care data available through the CVD-COVID-UK/COVID-IMPACT Consortium presented a significant opportunity to evaluate associations between mental disorder and mortality, our analysis of the impact of COVID-19 on these associations was limited since complete primary care data was only available from November 2019. With only four months of pre-pandemic data, we could not robustly account for seasonal fluctuations in mortality. Due to small numbers of people with schizophrenia and bipolar disorder, we combined the three mental disorders for the analyses of interaction with the time period. However, given the much higher prevalence of depression, this may have masked any variation in findings between disorders and we cannot conclusively rule out possible differences in mortality by time period for specific mental disorders.

### Implications

The attenuation of mortality estimates upon adjustment for receipt of care standards suggests that disparities in MI care may be a modifiable contributory factor to mental disorder disparities in one-year survival. The residual excess risk also indicates that other factors play a role. Future research should focus on quantifying the contribution of acute care and other potential contributing factors, including post-discharge care, using appropriate mediation analysis approaches, to inform understanding of mechanisms and, in turn, the development of tailored strategies. In-depth service evaluation, including interrogation of granular health data on MI symptom presentation, acute care pathways, and in-hospital transitions, is needed to identify the drivers of disparities in guideline-indicated acute care for MI. Future studies should also examine the contribution of lifestyle factors, such as smoking, BMI, physical activity, and alcohol use, to the mortality disparities. It would also be valuable to further examine mortality disparities for depression, accounting for the severity of depression and timing of depression episodes by incorporating other data, such as prescriptions. Qualitative studies of acute care for MI that include both the experiences of people with a mental disorder and an MI, and the experiences of the health care professionals who treat them, are also needed (with one such study from the authors of this paper underway). Given the established sociodemographic inequalities in cardiac care,^53^ analysis of the intersection between mental disorders and factors such as sex, deprivation, and ethnicity is also warranted.

Meanwhile, health professionals involved in the delivery of acute cardiac care to people with mental disorders should be cognizant of the higher risk of MI in this group, the potential for sub-optimal treatment of MI, and the contribution of this to excess mortality in the short term. Better integration of care across clinical specialities during acute MI care, including emergency care, cardiology, and liaison psychiatry, would support higher quality care. Together with improved continuity of care across secondary and primary care, and improved medication review and cardiac rehabilitation referral and access processes, this could lead to reductions in the entrenched MI mortality disparities experienced by this vulnerable sub-population.

## Conclusions

We found that, in England, 30-day and one-year mortality post-MI are generally higher in people with each of schizophrenia, bipolar disorder, and, to a lesser extent, depression. These disparities did not worsen during and subsequent to the COVID-19 pandemic. Differences in receipt of care may contribute to poorer one-year survival, reinforcing the urgent need for delivery of optimal acute cardiac care to people with a mental disorder.

## Supplementary Material

qcag044_Supplementary_Data

## Data Availability

The protocol, code lists and data curation and analysis code for this study are available on GitHub (https://github.com/BHFDSC/CCU046_02/). The data used in this study are available in NHS England’s Secure Data Environment (SDE) service for England, but as restrictions apply they are not publicly available (https://digital.nhs.uk/services/secure-data-environment-service). The CVD-COVID-UK/COVID-IMPACT programme, led by the BHF Data Science Centre (https://bhfdatasciencecentre.org/), received approval to access data in NHS England’s SDE service for England from the Independent Group Advising on the Release of Data (IGARD) (https://digital.nhs.uk/about-nhs-digital/corporate-information-and-documents/independent-group-advising-on-the-release-of-data) via an application made in the Data Access Request Service (DARS) Online system (ref. DARS-NIC-381078-Y9C5K) (https://digital.nhs.uk/services/data-access-request-service-dars/dars-products-and-services). The CVD-COVID-UK/COVID-IMPACT Approvals & Oversight Board (https://bhfdatasciencecentre.org/areas/cvd-covid-uk-covid-impact/) subsequently granted approval to this project to access the data within NHS England’s SDE service for England. The anonymous data used in this study were made available to accredited researchers only. Those wishing to gain access to the data should contact bhfdsc@hdruk.ac.uk in the first instance.
