# Supplementary material for: Mental disorders, mortality following myocardial infarction, and the impact of the COVID-19 pandemic in England: a cohort study
Source: Eur Heart J Qual Care Clin Outcomes. 2026 Mar 17;12(5):833–48. doi: 10.1093/ehjqcco/qcag044 (PMC13457930; doi:10.1093/ehjqcco/qcag044)
Supplement: Supplementary file 1 [file qcag044_supplementary_data.docx]

**Mental disorders, mortality following myocardial infarction and the impact of the COVID-19 pandemic in England: a cohort study**

**Supplementary Material**

Kelly Fleetwood^1^, John Nolan^2^, Colin Berry^3^, Debbie Cavers^1^, Stewart W Mercer^1^, Sandosh Padmanabhan^3^, Daniel J Smith^4^, Robert Stewart^5,6^, Amanda Vettini^1^, Caroline A Jackson^1^, on behalf of the CVD-COVID-UK/COVID-IMPACT Consortium

^1^Usher Institute, University of Edinburgh, Edinburgh, UK

^2^British Heart Foundation Data Science Centre, Health Data Research UK, London, UK

^3^School of Cardiovascular and Metabolic Health, University of Glasgow, Glasgow, UK

^4^Institute of Neuroscience and Cardiovascular Research, University of Edinburgh, UK

^5^Department of Psychological Medicine, King’s College London, London, UK

^6^South London and Maudsley NHS Foundation Trust, London, UK

Correspondence to:

Kelly Fleetwood

Email: [kelly.fleetwood@ed.ac.uk](mailto:kelly.fleetwood@ed.ac.uk)

Address: Usher Institute, Usher Building, The University of Edinburgh, 5‒7 Little France Road, Edinburgh BioQuarter ‒ Gate 3, Edinburgh, EH16 4UX, UK

Contents

[Methods S1: SNOMED code lists for mental disorders	3](#_Toc219377467)

[Methods S2: Additional description of variables	4](#_Toc219377468)

[Methods S3: Covariates included in each statistical model	8](#_Toc219377469)

[Figure S1: Flow diagram for the analysis cohorts	8](#_Toc219377470)

[Figure S2: Odds ratios and hazard ratios for mortality following NSTEMI in patients with schizophrenia, bipolar disorder or depression versus those without any of these disorders. Models 1 to 5.	10](#_Toc219377471)

[Figure S3: Odds ratios and hazard ratios for mortality following STEMI in patients with schizophrenia, bipolar disorder or depression versus those without any of these disorders. Models 1 to 5.	11](#_Toc219377472)

[Figure S4: Odds ratios for receipt of each care standard following NSTEMI in patients with schizophrenia, bipolar disorder or depression versus those without any of these disorders. Sensitivity analysis. Models 1 to 4.	12](#_Toc219377473)

[Figure S5: Odds ratios for receipt of each care standard following STEMI in patients with schizophrenia, bipolar disorder or depression versus those without any of these disorders. Sensitivity analysis. Models 1 to 4.	13](#_Toc219377474)

[Figure S6: MI admissions and mortality by admission month	14](#_Toc219377475)

[Figure S7: MI admissions and mortality by 4-month admission period and mental disorder	15](#_Toc219377476)

[Table S1: Interaction between mental disorder and 4-month time period; likelihood ratio tests of whether adding the interaction improved the fit of the model (primary analysis)	16](#_Toc219377477)

[Table S2: Hazard ratios by 4-month time period for time to CVD mortality within the first year following NSTEMI in patients with versus without any of schizophrenia, bipolar disorder or depression. Model 5 with interaction between mental disorder and 4-month time period	16](#_Toc219377478)

[Table S3: Interaction between mental disorder and 4-month time period; likelihood ratio tests of whether adding the interaction improved the fit of the sensitivity analysis models	17](#_Toc219377479)

[Table S4: Hazard ratios by 4-month time period for time to CVD mortality within the first year following NSTEMI in patients with versus without any of schizophrenia, bipolar disorder or depression. Model 4 with interaction between mental disorder and 4-month time period. Sensitivity analysis	17](#_Toc219377480)

# Methods S1: SNOMED code lists for mental disorders

The General Practice Extraction Service Data for Pandemic Planning and Research (GDPPR) dataset includes a subset of all SNOMED codes used in the United Kingdom. The subset of codes included is defined by the primary care domain (PCD) reference set.^1^ The PCD reference set is also used by the Quality and Outcomes Framework (QOF) and current and historical versions of the reference set are available from NHS England.^2^ The available SNOMED codes are grouped into code clusters.^1^

To develop our SNOMED code lists for mental disorders we extracted codes from all of the clusters related to relevant mental disorder diagnoses: DEPR_COD (depression diagnosis codes), DEPRES_COD (depression resolved codes) and MH_COD (psychosis and schizophrenia and bipolar affective disease codes) from version 47.1 of the QOF cluster list (which was the most recent version at the time of developing the SMI code lists).^3^ Additionally, we mapped existing Read v2 and Clinical Terms Version 3 code lists^4^ for schizophrenia, bipolar disorder and depression to SNOMED, and identified codes available within the GDPPR dataset. We collated all SNOMED codes identified from the clusters, and from the mapping process and then identified codes for each of schizophrenia, bipolar disorder or depression.

All of our SNOMED code lists are available via the HDR UK Phenotype Library:

- **Schizophrenia:** <https://phenotypes.healthdatagateway.org/phenotypes/PH1718>
- **Bipolar disorder:** <https://phenotypes.healthdatagateway.org/phenotypes/PH1719>
- **Depression:** <https://phenotypes.healthdatagateway.org/phenotypes/PH1720>

## References

1. NHS Digital. *General Practice Extraction Service (GPES) Data for Pandemic Planning and Research (GDPPR): a guide for analysts and users of the data.* 2024. <https://digital.nhs.uk/coronavirus/gpes-data-for-pandemic-planning-and-research/guide-for-analysts-and-users-of-the-data> (13 Jan 2025, date last accessed).
2. NHS Digital. *Quality and Outcomes Framework (QOF) business rules*. 2024. <https://digital.nhs.uk/data-and-information/data-collections-and-data-sets/data-collections/quality-and-outcomes-framework-qof/business-rules> (13 Jan 2025, date last accessed)
3. NHS Digital. *Quality and Outcomes Framework (QOF) business rules v47.0 2022-2023*. 2023. <https://digital.nhs.uk/data-and-information/data-collections-and-data-sets/data-collections/quality-and-outcomes-framework-qof/quality-and-outcome-framework-qof-business-rules/quality-and-outcomes-framework-qof-business-rules-v47.0-2022-2023> (13 Jan 2025, date last accessed).
4. Prigge R, Fleetwood KJ, Jackson CA, Mercer S, Kelly PAT, Sudlow C *et al*. Robustly Measuring Multiple Long-Term Health Conditions Using Disparate Linked Datasets in UK Biobank. Available at SSRN: <https://ssrn.com/abstract=4863974>.

# Methods S2: Additional description of variables

## Myocardial infarction type

MINAP provides the following recommended definitions of STEMI and NSTEMI in their guide to the MINAP dataset.^1^

### STEMI

“There will normally be a history consistent with the diagnosis. The diagnosis requires the presence of cardiographic changes of ST elevation consistent with infarction of ≥2mm in contiguous chest leads and/or ST elevation of ≥1mm ST elevation in 2 or more standard leads. (New LBBB [left bundle branch block] is included.) There must be enzyme or troponin elevation. Where CK [creatine kinase] is used the peak value should exceed twice the upper limit of the reference range. Where troponin assay is used the locally accepted cut off value should be used.”

“This group includes all patients with STEMI regardless of whether typical changes were evident on the admission of ECG or developed subsequently.”

### NSTEMI

“There must be symptoms consistent with cardiac ischaemia, and there will normally be cardiographic changes consistent with this diagnosis. Troponin elevation above the locally determined reference level is mandatory.”

## Admission timing

We used the MINAP hospital arrival time variable to determine whether the admission was during the day (6am – 6pm) or overnight.

## Admission day

We used the MINAP hospital arrival time variable to determine whether the admission was on a weekday or weekend.

## 4-month period of admission

We used the MINAP hospital arrival time variable to identify the 4-month period during which the MI admission occurred, starting from the pre-pandemic period of November 2019 to February 2020.

## Hospital

We ascertained the hospital the patient was admitted to by linking the MINAP record to HES APC records.

## Comorbid angina

We identified comorbid angina if any of the following occurred:

- The MINAP previous angina field (field 2.06) recorded “1. Yes”
- The patient had a diagnosis of angina recorded in GDPPR prior to their date of MI admission.
- The patient had a diagnosis of angina recorded in HES APC prior to their date of MI admission.

We identified diagnoses using existing SNOMED (GDPPR) and ICD-10 (HES APC) code lists for angina which are available on the HDR UK Phenotype Library.^2^

## Comorbid cerebrovascular disease

We identified comorbid cerebrovascular disease if any of the following occurred:

- The MINAP cerebrovascular disease field (field 2.10) recorded “1. Yes”
- The patient had a diagnosis of cerebrovascular disease recorded in GDPPR prior to their date of MI admission. We identified diagnoses using SNOMED codes in the haemorrhagic stroke (HSTRK_COD), non-haemorrhagic stroke (OSTR_COD), stroke diagnosis (STRK_COD) and transient ischaemic attack (TIA_COD) code clusters (see Methods S2 for further explanation of code clusters) and additionally the SNOMED codes 195163003, 67992007 and 73192008.
- The patient had a diagnosis of cerebrovascular disease recorded in HES APC prior to their date of MI admission. We identified diagnoses using ICD-10 codes I60-I69 and G45.

Our code lists for comorbid cerebrovascular disease are available on our GitHub repository (<https://github.com/BHFDSC/CCU046_01>) and via the HDR UK Phenotype Library: <https://phenotypes.healthdatagateway.org/phenotypes/PH1791>.

## Comorbid chronic renal failure

We identified comorbid chronic renal failure if any of the following occurred:

- The MINAP chronic renal failure field (field 2.12) recorded “1. Yes”
- The patient had a diagnosis of chronic renal failure recorded in GDPPR prior to their date of MI admission. We identified diagnoses using SNOMED codes. We developed the code list by identifying chronic renal failure codes from an existing chronic kidney disease code list^3^ and the GDPPR code cluster for chronic kidney disease stage 4 and 5 (CKDATRISK1_COD).
- The patient had a diagnosis of chronic renal failure recorded in HES APC prior to their date of MI admission. We identified diagnoses using ICD-10 codes. We developed the code list by identifying chronic renal failure codes from an existing chronic kidney disease code list^3^ and existing end stage renal disease code lists.^4,5^

Our code lists for comorbid chronic renal failure are available on our GitHub repository (<https://github.com/BHFDSC/CCU046_01>) and via the HDR UK Phenotype Library: <https://phenotypes.healthdatagateway.org/phenotypes/PH1792>.

## Comorbid diabetes

We identified comorbid diabetes if any of the following occurred:

- The MINAP diabetes field (field 2.17) recorded "1. Diabetes (dietary control)" or "2. Diabetes (oral medicine)" or "3. Diabetes (insulin)" or "5. Insulin plus oral medication"
- The patient had a diagnosis of diabetes recorded in GDPPR prior to their date of MI admission.
- The patient had a diagnosis of diabetes recorded in HES APC prior to their date of MI admission.

We identified diagnoses using existing SNOMED (GDPPR) and ICD-10 (HES APC) code lists for diabetes which are available on the HDR UK Phenotype Library.^6^

## Comorbid heart failure

We identified comorbid heart failure if any of the following occurred:

- The MINAP heart failure field (field 2.13) recorded “1. Yes”
- The patient had a diagnosis of heart failure recorded in GDPPR prior to their date of MI admission.
- The patient had a diagnosis of heart failure recorded in HES APC prior to their date of MI admission.

We identified diagnoses using existing SNOMED (GDPPR) and ICD-10 (HES APC) code lists for heart failure which are available on the HDR UK Phenotype Library.^7^

## Previous MI

We identified previous MI if either of the following occurred:

- The patient had a diagnosis of MI recorded in GDPPR more than 3 days prior to their date of MI admission (to ensure that we didn’t count the index event). We identified diagnoses by adapting an existing MI SNOMED code list.^8^
- The patient had a diagnosis of MI recorded in HES APC more than 3 days prior to their date of MI admission. We identified diagnoses using ICD-10 codes (I21, I22) as per previous studies from the authors.^9^

Our code lists for myocardial infarction are available on our GitHub repository (<https://github.com/BHFDSC/CCU046_01>) and via the HDR UK Phenotype Library (<https://phenotypes.healthdatagateway.org/phenotypes/PH1722>).

We couldn’t use the MINAP previous MI field (field 2.05) because it wasn’t included in the version of the MINAP dataset held by the CVD-COVID-UK/COVID-IMPACT Consortium at the time of data curation.

## Previous PCI

We identified previous PCI if any of the following occurred:

- The MINAP previous PCI field (field 2.18) recorded “1. Yes”
- The patient had a PCI recorded in HES APC prior to their date of MI admission. We identified PCI using the OPCS-4 procedure code K75, in line with previous studies from the authors.^9^

We didn’t use GDPPR to identify PCI because the GDPPR clusters do not include a comprehensive set of PCI codes.

## COVID-19 positive

We identified patients who had COVID-19 within 14 days either side of their MI admission based on diagnoses recorded in GDPPR or HES APC, hospitalised cases of COVID-19 recorded in CHESS and positive tests for COVID-19 recorded in SGSS or SUS.

## Guideline informed care standards

### Receipt of angiography (NSTEMI)

We categorised receipt of angiography as:

- Received within 72 hours
- Received out with 72 hours
- Received, timing unknown
- Eligible but did not receive
- Not eligible

We derived this variable from the MINAP fields for coronary angiography (field 4.13), time of arrival at hospital (field 3.06) and time of local angiography (field 4.18).

### Admission to cardiac ward (NSTEMI)

Admission to cardiac ward was included as a binary variable, defined as ‘yes’ if the MINAP admission ward field (field 3.17) was recorded as ‘cardiac care unit’, ‘intensive therapy unit’ or ‘cardiac ward (non CCU)’ and otherwise ‘no’ if the field was not recorded as unknown or left blank.

### Receipt of primary PCI (STEMI)

We defined a binary receipt of PCI variable from the MINAP field initial reperfusion treatment (field 3.39).

### Call-to-balloon (CTB) time within target (STEMI)

The MINAP report defines two targets for CTB times: 120 minutes and 150 minutes. We categorised CTB time as:

- Less than 120 minutes
- Between 120 and 150 minutes
- Greater than or equal to 150 mins
- Received pPCI but CTB time unknown
- Did not receive pPCI

We derived this variable from the MINAP fields initial reperfusion treatment (field 3.39), time of call for help (field 3.02) and time of reperfusion treatement (field 3.09).

### Indicated secondary prevention medication prescribed at discharge (both types of MI)

Indicated secondary prevention medication prescribed at discharge was included as a binary variable, defined as ‘yes’ if where indicated, the patient was prescribed (or refused) aspirin (field 4.08), beta blocker (field 4.05), statin (field 4.07), either ACE inhibitor or angiotensin receptor antagonist (field 4.06), and either thienopyridine (field 4.27) or ticagrelor (field 4.31).

### Referral for cardiac rehabilitation (both types of MI)

We categorised referral for cardiac rehabilitation as follows, based on the MINAP cardiac rehabilitation field (field 4.09)

- Yes
- No
- Not indicated
- Patient declined

## References

1. National Institute for Cardiovascular Outcomes Research (NICOR). Myocardial Ischaemia National Audit Project Dataset. 2024. <https://www.nicor.org.uk/publications/data-sets/heart-attack-minap/minap-dataset-v10-3-2> (14 January 2026, date last accessed).
2. Knight R, Walker V, Ip S, Cooper JA, Bolton T, Keene S et al. *PH956 / 2134 - CCU002_01 Angina.* 2022. [http://phenotypes.healthdatagateway.org/phenotypes/PH956/version/2134/detail/](http://phenotypes.healthdatagateway.org/phenotypes/PH956/version/2134/detail/%20) (24 April 2025, date last accessed).
3. Knight R, Walker V, Ip S, Cooper JA, Bolton T, Keene S et al. *PH950 / 2128 - CCU002_01 Chronic Kidney Disease (CKD).* 2022. <http://phenotypes.healthdatagateway.org/phenotypes/PH950/version/2128/detail/> (25 April 2025, date last accessed).
4. Mansfield KE, Nitsch D, Smeeth L, Bhaskaram K, Tomlinson LA. *PH574 / 1148 - End-Stage Renal Disease (ESRD).* 2021. <http://phenotypes.healthdatagateway.org/phenotypes/PH574/version/1148/detail/> (25 April 2025, date last accessed).
5. Prigge R, Fleetwood KJ, Jackson CA, Mercer S, Kelly PAT, Sudlow C *et al*. Robustly Measuring Multiple Long-Term Health Conditions Using Disparate Linked Datasets in UK Biobank. Available at SSRN: <https://ssrn.com/abstract=4863974>.
6. Wood A, Denholm R, Hollings S, Cooper J, Ip S, Walker V et al. *PH945 / 2123 - CCU000 Diabetes.* 2022. <http://phenotypes.healthdatagateway.org/phenotypes/PH945/version/2123/detail/> (25 April 2025, date last accessed).
7. Knight R, Walker V, Ip S, Cooper JA, Bolton T, Keene S et al. *PH968 / 2146 - CCU002_01 Heart failure.* 2022. [http://phenotypes.healthdatagateway.org/phenotypes/PH968/version/2146/detail/](http://phenotypes.healthdatagateway.org/phenotypes/PH968/version/2146/detail/.%20) (25 April 2025, date last accessed).
8. Wood A, Denholm R, Hollings S, Cooper J, Ip S, Walker V et al. *PH942 / 2120 - CCU000 Acute Myocardial Infarction (AMI)*. 2022. <http://phenotypes.healthdatagateway.org/phenotypes/PH942/version/2120/detail/> (25 April 2025, date last accessed).
9. Fleetwood K, Wild SH, Smith DJ, Mercer SW, Licence K, Sudlow CLM, et al. Severe mental illness and mortality and coronary revascularisation following a myocardial infarction: a retrospective cohort study. *BMC Med* 2021;**19**:67. doi: <https://doi.org/10.1186/s12916-021-01937-2>

# Methods S3: Covariates included in each statistical model

The table below lists the covariates added to each subsequent model, such that model 1 includes the covariates listed for model 1, model 2 includes the covariates listed for models 1 and 2, and so on. The covariates are defined in the manuscript in the Covariates subsection of the Methods, and above in Methods S2.

|  | **Covariates added to each model** | |
| --- | --- | --- |
| **Model** | **NSTEMI** | **STEMI** |
| 1 | Hospital (random effect for the intercept) | Hospital (random effect for the intercept) |
|  | Age | Age |
|  | Sex | Sex |
| 2 | Ethnicity | Ethnicity |
|  | Deprivation | Deprivation |
| 3 | **Timing variables**   - Daytime/overnight MI admission | **Timing variables**   - Daytime/overnight MI admission |
|  | - Weekday/weekend MI admission | - Weekday/weekend MI admission |
|  | - 4-month period during which the MI admission occurred | - 4-month period during which the MI admission occurred |
| 4 | **Clinical history**   - History of MI | **Clinical history**   - History of MI |
|  | - History of percutaneous coronary intervention | - History of percutaneous coronary intervention |
|  | - Comorbid angina | - Comorbid angina |
|  | - Comorbid cerebrovascular disease | - Comorbid cerebrovascular disease |
|  | - Comorbid chronic renal failure | - Comorbid chronic renal failure |
|  | - Comorbid diabetes | - Comorbid diabetes |
|  | - Comorbid heart failure | - Comorbid heart failure |
| 5 | **MI presentation features** | **MI presentation features** |
|  | - Cardiac arrest | - Cardiac arrest |
|  | - Cardiogenic shock | - Cardiogenic shock |
|  | - Creatinine | - Creatinine |
|  | - Heart rate | - Heart rate |
|  | - Systolic blood pressure | - Systolic blood pressure |
|  | - COVID-19 status | - COVID-19 status |
|  | - ST deviation |  |

MI, myocardial infarction

For one-year mortality amongst patients discharged home, we additionally adjusted for guideline-informed care standards. In the primary analysis, the care standards were added to model 5, and in the sensitivity analysis the care standards were added to model 4. We have labelled the additional sets of covariates a to d, such that, model 5a includes all the variables in model 5, plus the care standards in set a, and so on. Note that care standards for NSTEMI were added sequentially, whereas for STEMI set a included receipt of primary percutaneous coronary intervention (PCI) (yes/no), which was replaced in set b by an alternative covariate which also captured the timing of PCI: call-to-balloon time within target (<120 mins, 120–150 mins, ≥150 mins, received PCI but time unknown, did not receive PCI).

|  | **Sets of care standards added to models** | |
| --- | --- | --- |
| **Set** | **NSTEMI** | **STEMI** |
| a | - Receipt of angiography | - Receipt of primary percutaneous coronary intervention |
| b | - Receipt of angiography - Referral for cardiac rehabilitation | - Call-to-balloon time within target |
| c | - Receipt of angiography - Referral for cardiac rehabilitation - Indicated secondary prevention medication prescribed at discharge | - Call-to-balloon time within target - Referral for cardiac rehabilitation |
| d | - Receipt of angiography - Referral for cardiac rehabilitation - Indicated secondary prevention medication prescribed at discharge - Admission to cardiac ward | - Call-to-balloon time within target - Referral for cardiac rehabilitation - Indicated secondary prevention medication prescribed at discharge |

# Figure S1: Flow diagram for the analysis cohorts

Cohort of patients with NSTEMI or STEMI

N = 210 120

1-year mortality amongst patients discharged home

N = 98 830 (patients discharged home)

N^1^ = 73 995 (74.9%)

N^2^ = 84 960 (86.0%)

1-year mortality amongst patients discharged home

N = 66 810 (patients discharged home)

N^1^ = 53 020 (79.4%)

N^2^ = 62 220 (93.1%)

1-year mortality amongst patients surviving 30 days

N = 124 480 (patients surviving 30 days)

N^1^ = 102 480 (82.3%)

N^2^ = 120 945 (97.2%)

30-day mortality

1-year mortality

Time to all-cause mortality within the first year

Time to CVD mortality within the first year

N = 79 045

N^1^ = 62 795 (79.4%)

N^2^ = 77 545 (98.1%)

1-year mortality amongst patients surviving 30 days

N = 71 605 (patients surviving 30 days)

N^1^ = 57 875 (80.8%)

N^2^ = 70 495 (98.4%)

30-day mortality

1-year mortality

Time to all-cause mortality within the first year

Time to CVD mortality within the first year

N = 131 075

N^1^ = 107 550 (82.1%)

N^2^ = 127 220 (97.1%)

N = patients eligible for inclusion in analysis

N^1^ = complete cases (with percentage of N)

N^2^ = complete cases (sensitivity analysis) (with percentage of N)

As per NHSE’s SDE statistical disclosure control rules, counts are rounded to the nearest 5 and counts less than 10 are suppressed

Patients with NSTEMI

N = 131 075

Patients with STEMI

N = 79 045

# Figure S2: Odds ratios and hazard ratios for mortality following NSTEMI in patients with schizophrenia, bipolar disorder or depression versus those without any of these disorders. Models 1 to 5.

Model 1 adjusts for age, sex and hospital (random effect); model 2 additionally adjusts for ethnicity and area-level deprivation; model 3 adds timing variables; model 4 adds clinical history; model 5 adds MI presentation features; model 5a adds receipt of angiography; 5b adds referral for cardiac rehabilitation; 5c adds indicated secondary prevention medication prescribed at discharge and 5d adds admission to cardiac ward.

^a^1.062 (1.004, 1.123)

# Figure S3: Odds ratios and hazard ratios for mortality following STEMI in patients with schizophrenia, bipolar disorder or depression versus those without any of these disorders. Models 1 to 5.

Model 1 adjusts for age, sex and hospital (random effect); model 2 additionally adjusts for ethnicity and area-level deprivation; model 3 adds timing variables; model 4 adds clinical history; model 5 adds MI presentation features; model 5a adds receipt of primary PCI (yes/no); in model 5b the binary primary PCI is replaced by the categorical CTB time within target variable (<120 mins, 120–150 mins, ≥150 mins, received PCI but time unknown, did not receive PCI); 5c adds referral for cardiac rehabilitation and 5d adds indicated secondary prevention medication prescribed at discharge.

^a^1.078 (1.001, 1.161); ^b^1.073 (0.996	, 1.155); ^c^1.331 (1.005, 1.762)

# Figure S4: Odds ratios for receipt of each care standard following NSTEMI in patients with schizophrenia, bipolar disorder or depression versus those without any of these disorders. Sensitivity analysis. Models 1 to 4.

Model 1 adjusts for age, sex and hospital (random effect); model 2 additionally adjusts for ethnicity and area-level deprivation; model 3 adds timing variables; model 4 adds clinical history; model 4a adds receipt of angiography; 4b adds referral for cardiac rehabilitation; 4c adds indicated secondary prevention medication prescribed at discharge and 4d adds admission to cardiac ward.

# Figure S5: Odds ratios for receipt of each care standard following STEMI in patients with schizophrenia, bipolar disorder or depression versus those without any of these disorders. Sensitivity analysis. Models 1 to 4.

Model 1 adjusts for age, sex and hospital (random effect); model 2 additionally adjusts for ethnicity and area-level deprivation; model 3 adds timing variables; model 4 adds clinical history; model 4a adds receipt of primary PCI (yes/no); in model 4b the binary primary PCI is replaced by the categorical CTB time within target variable (<120 mins, 120–150 mins, ≥150 mins, received PCI but time unknown, did not receive PCI); 4c adds referral for cardiac rehabilitation and 4d adds indicated secondary prevention medication prescribed at discharge.

^a^1.348 (1.002, 1.812), ^b^1.5685 (1.0001, 2.4598)

# Figure S6: MI admissions and mortality by admission month

# Figure S7: MI admissions and mortality by 4-month admission period and mental disorder

# Table S1: Interaction between mental disorder and 4-month time period; likelihood ratio tests of whether adding the interaction improved the fit of the model (primary analysis)

| MI type | Outcome | $\chi_{9}^{2}$ | p-value |
| --- | --- | --- | --- |
| NSTEMI | 30-day mortality | 6.614 | 0.677 |
|  | 1-year mortality | 10.086 | 0.344 |
|  | 1-year mortality (patients surviving 30 days) | 9.182 | 0.421 |
|  | 1-year mortality (patients discharged home) | 9.582 | 0.385 |
|  | Time to mortality within the first year | 10.893 | 0.283 |
|  | Time to CVD mortality within the first year | 17.198 | 0.046 |
| STEMI | 30-day mortality | 4.394 | 0.884 |
|  | 1-year mortality | 4.920 | 0.841 |
|  | 1-year mortality (patients surviving 30 days) | 8.984 | 0.439 |
|  | 1-year mortality (patients discharged home) | 9.779 | 0.369 |
|  | Time to mortality within the first year | 5.385 | 0.800 |
|  | Time to CVD mortality within the first year | 2.969 | 0.966 |

# Table S2: Hazard ratios by 4-month time period for time to CVD mortality within the first year following NSTEMI in patients with versus without any of schizophrenia, bipolar disorder or depression. Model 5 with interaction between mental disorder and 4-month time period

| 4-month time period | Hazard ratio (95% confidence interval) |
| --- | --- |
| Nov 2019 - Feb 2020 | 1.15 (0.99, 1.35) |
| Mar 2020 - Jun 2020 | 1.05 (0.88, 1.26) |
| Jul 2020 - Oct 2020 | 1.16 (0.98, 1.38) |
| Nov 2020 - Feb 2021 | 0.83 (0.69, 0.99) |
| Mar 2021 - Jun 2021 | 1.10 (0.94, 1.28) |
| Jul 2021 - Oct 2021 | 1.10 (0.93, 1.30) |
| Nov 2021 - Feb 2022 | 0.95 (0.80, 1.12) |
| Mar 2022 - Jun 2022 | 1.27 (1.07, 1.51) |
| Jul 2022 - Oct 2022 | 1.14 (0.95, 1.36) |
| Nov 2022 - Feb 2023 | 0.98 (0.82, 1.17) |

# Table S3: Interaction between mental disorder and 4-month time period; likelihood ratio tests of whether adding the interaction improved the fit of the sensitivity analysis models

| MI type | Outcome | $\chi_{9}^{2}$ | p-value |
| --- | --- | --- | --- |
| NSTEMI | 30-day mortality | 12.772 | 0.173 |
|  | 1-year mortality | 10.205 | 0.334 |
|  | 1-year mortality (patients surviving 30 days) | 9.091 | 0.429 |
|  | 1-year mortality (patients discharged home) | 9.727 | 0.373 |
|  | Time to mortality within the first year | 11.230 | 0.260 |
|  | Time to CVD mortality within the first year | 17.547 | 0.041 |
| STEMI | 30-day mortality | 5.710 | 0.769 |
|  | 1-year mortality | 5.323 | 0.805 |
|  | 1-year mortality (patients surviving 30 days) | 8.278 | 0.506 |
|  | 1-year mortality (patients discharged home) | 11.954 | 0.216 |
|  | Time to mortality within the first year | 4.841 | 0.848 |
|  | Time to CVD mortality within the first year | 4.956 | 0.838 |

# Table S4: Hazard ratios by 4-month time period for time to CVD mortality within the first year following NSTEMI in patients with versus without any of schizophrenia, bipolar disorder or depression. Model 4 with interaction between mental disorder and 4-month time period. Sensitivity analysis

| 4-month time period | Hazard ratio (95% confidence interval) |
| --- | --- |
| Nov 2019 - Feb 2020 | 1.07 (0.93, 1.24) |
| Mar 2020 - Jun 2020 | 1.01 (0.86, 1.18) |
| Jul 2020 - Oct 2020 | 1.17 (1.00, 1.36) |
| Nov 2020 - Feb 2021 | 0.85 (0.72, 1.00) |
| Mar 2021 - Jun 2021 | 1.16 (1.00, 1.33) |
| Jul 2021 - Oct 2021 | 1.04 (0.90, 1.21) |
| Nov 2021 - Feb 2022 | 0.96 (0.83, 1.12) |
| Mar 2022 - Jun 2022 | 1.21 (1.04, 1.40) |
| Jul 2022 - Oct 2022 | 1.15 (0.98, 1.34) |
| Nov 2022 - Feb 2023 | 0.96 (0.82, 1.12) |
